# What and how are students taught about communicating risks to patients? Analysis of a medical curriculum

**DOI:** 10.1371/journal.pone.0233682

**Published:** 2020-05-29

**Authors:** Franziska Baessler, Joshua Weidlich, Sophie Schweizer, Anja Ciprianidis, Marina Bartolovic, Ali Zafar, Michael Wolf, Fabienne Louise Wagner, Tabea Chiara Baumann, André L. Mihaljevic, Beate Ditzen, Daniela Roesch-Ely, Christoph Nikendei, Jobst-Hendrik Schultz

**Affiliations:** 1 Department of General, Internal and Psychosomatic Medicine, Center for Psychosocial Medicine, Heidelberg University Hospital, Heidelberg, Germany; 2 Department of Gynecology and Obstetrics, Heidelberg University Hospital, Heidelberg, Germany; 3 Department of General Psychiatry, Center for Psychosocial Medicine, Heidelberg University Hospital, Heidelberg, Germany; 4 Department of General, Visceral and Transplantation Surgery, University Hospital Heidelberg, Heidelberg, Germany; 5 Institute of Medical Psychology, Heidelberg University Hospital, Heidelberg, Germany; King Saud University, SAUDI ARABIA

## Abstract

**Background:**

Communication is a core competence in medical care. Failure of physicians to properly communicate inherent risks of medical interventions has been linked with inadequate training at school. This study analyses a medical curriculum for assessing the content and quality of teaching risk communication to students.

**Methods:**

A checklist based on the national guidelines of core competencies on risk communication required of physicians was developed. Participant observers surveyed all teaching sessions at a medical school during a semester to record the frequency, characteristics and clinical context used by lectures during classes. Data were analyzed using statistical and descriptive methods to determine the prevalence and quality of teaching content.

**Results:**

231 teaching sessions were surveyed. The inter-rater reliability was 81%. Lecturers mentioned topics of risk communication in 61.5% of teaching sessions (83.7% in surgery, 43.3% in internal medicine) but core biostatistics concepts were not discussed in more than 80% of these sessions. Important topics such as patient safety and preventable diseases were underrepresented. Risk communication was mainly taught in large-group, theoretical sessions and rarely with supplementary teaching material (7.4%). Students asked questions in 15.2% of courses, more often in surgery classes than in internal medicine.

**Conclusion:**

Statistical and clinical topics relevant for teaching risk communication to medical students are not only underrepresented but also minimally explained by lecturers. Supplementary material on risk communication is rarely provided to students during classes. High-resource demanding, small-group teaching formats are not necessarily interactive as students ask few questions.

## Introduction

Medical treatments are inherently risky and the precise nature of the risks involved for any given intervention needs to be communicated effectively to patients. For reaching shared, informed decisions, physician-patient interactions must include a summary of the associated risks, probabilities of occurrence and their consequences not only for suggested treatments of diagnosed ailments but also about prospective illnesses [1]. This risk communication is defined as “…the open two-way exchange of information and opinion about harms and benefits, with the aim of improving the understanding of risk and of promoting better decisions about clinical management” [2].

Efficient physician-patient communication has been associated with improved health outcomes, shared decision-making and therapy adherence [3–8], whereas miscommunication with physicians is a leading cause for medical lawsuits by patients [9]. In Germany, such claims are mostly related to orthopedics and surgery procedures (42%, 6,363 cases per year) and internal and general medicine (12%, 1,762 cases per year) [10]. Previous studies show that many physicians have problems in communicating risks and risk values correctly and comprehensibly [10–12]. Two major reasons for the difficulties physicians experience in this context are poor teaching of statistical concepts at medical schools and a general lack of communication skills [11–16]. This may have serious consequences as biased risk perception is associated with problematic health behavior and negative health outcomes [17–19].

Given the growing awareness about consequences within the context of shared decision-making [20–23], medical schools have introduced biostatistics and communication courses to enhance understanding of risk communication among students [24–27]. These courses may be taught in different settings; in theoretical sessions such as statistical explanations, and in practical contexts such as clinical cases or simulated patients [28, 29]. Likewise, University of Heidelberg–Germany`s oldest university with one of the most competitive medical schools (8% acceptance rate in 2017/2018) [30, 31]–has also restructured its medical curriculum (HeiCuMed) incorporating risk communication courses into a competency-based, six-year program [32–34]. The modifications were based on the German medical education guidelines–the National Competency-based Learning Objectives Catalogue in Medicine (NKLM)–that defined the competency level and learning goals for risks communication for physicians [35]. The NKLM states that physicians should recognize healthy and risky behaviors of patients and communicate appropriately with them regarding errors and risks based on knowledge of counseling and therapy options [35]. Although efforts have been made to enhance students’ skills by implementing more effective biostatistics and communication courses at universities [24–26, 36], how thoroughly risk communication is taught to students at school remains unclear and has not been investigated previously.

This study aimed to assess the quantity and quality of teaching content on communicating risks and statistical values in the medical curriculum of two major disciplines at the University of Heidelberg’s medical school. The assessment consisted of the following principal questions: a) how often are students taught to communicate risks to patients (prevalence); which topics/vignettes are used to teach students on communicating risks (content), and how thoroughly is the topic taught by lecturers (quality)?

By surveying teaching sessions for a semester, we observed if and how students are taught about communicating risks; what topics and on what level are they used to teach students; if the sessions are interactive; and how often supplementary teaching aids are used by lecturers to deliver their message. These findings will help identify gaps in medical education about risk communication which serves as a prerequisite for training future doctors on risk communication.

## Methods

### Study design

This observational study was based on the concept of curriculum mapping, which has been employed by medical educators to observe how frequently and extensively students are taught a subject at school [36, 37]. All teaching sessions at the internal medicine and surgery departments of Heidelberg University Medical School were assessed by two participant observers on a day-to-day basis during the 2016/2017 winter semester. The teaching sessions consisted of large-group teaching formats such as lectures and seminars (where attendance is not mandatory), and small-group formats such as Medi-KIT, Bed-Side Teaching and Problem-Based Learning (PBL). Medi-KIT is a novel course wherein students practice communication skills with the help of simulated patients [38, 39], allowing them to take a medical history, educate patients or how to deliver bad news to them. In PBL sessions, students use “triggers” from a vignette to identify their own learning issues [29, 40]. In Skills Lab, students learn practical skills such as physical examination, suturing, and interpretation of X-rays [32, 41, 42]. The observers recorded the frequency and characteristics of content on risk communication using a checklist specifically designed for the study. By triangulating data on prevalence, content and intensity of teaching, we determined the quality and gaps in knowledge transfer of risk communication concepts in the curriculum.

### Checklist

A stepwise process based on the Association for Medical Education in Europe (AMEE) guides for designing high-quality questionnaires with particular emphasis on developing survey scales was employed [37, 43]. A thorough literature review revealed a relationship between physician-patient miscommunication and lack of relevant teaching at medical schools [14, 15]. In the second stage, medical educators and professors at Heidelberg medical school were interviewed regarding prospective curriculum gaps on risk communication.

A group of 15 experts of medical education, psychology, communication and biostatistics was formed to consult the NKLM as a reference guide. Several clauses in the NKLM, particularly 14c.4.2 [35], outline the competence criteria for physicians on communicating risks with patients, specifically mentioning knowledge about characteristic concepts such as survival/mortality rates, diagnostic measures and expected success, and statistical values such as specificity, sensitivity and p-value as key to better patient-physician communication outcomes. Using several NKLM clauses, particularly 14c, six learning outcomes for risk communication were identified and formulated into checklist items:

1. “Diagnostic and therapeutic measures” (NKLM 14c.4.2.1);
2. “Expected success, benefits, risks and costs” (14c.4.2.1)
3. “Positive and negative consequences” 14c.2.6.2);
4. “Uncertainty and evaluation models” (14c.4.2.3);
5. “Sources of misjudgment” (11.3.3.1); and
6. “Characteristic factors of diagnostic tests” (15.1.1).

A draft questionnaire was circulated among the team and finalized through Delphi rounds [44] in accordance with current best practices in survey designing. The final checklist included 19 core, descriptive and statistical concepts essential to teaching risk communication. It also included general information on the course such as the type of the teaching format and how the subject was taught while another section included questions on which topics were taught. The last three items were ancillary, open-ended comments for getting the participants’ personal view on teaching quality, what they liked about the class and how could it be improved.

This checklist was used by observers for recording the prevalence and content of teaching of these concepts i.e. whether or not the lecturer used these characteristic values and how descriptively were they taught. The prevalence of these variables was rated on a scale similar to Miller’s Pyramid [45] beginning from a lower rating a) not mentioned and b) mentioned, to the higher c) reflected upon, d) transferred to a clinical context, and the highest rating e) set in practical context. The ratings ‘c’ and above were considered intensive/in-depth teaching.

### Raters

To ensure that respondents interpreted the items in the intended manner, students of surgery and internal medicine departments in their clinical semesters were recruited as observers for the study. Fluency in German language was mandatory since the primary language of instruction at the medical school is German. Students were briefed about the study design, methodology and the aims of the project. Four students (male = 3, female = 1; aged between 23 and 28 years) were recruited on a semester-long contract for observing all classes. Consent to participate was included within the contract.

Two medical education experts trained the participants on completing the checklist based on their observations and understanding of the teaching content and methods employed by the lecturers to teach risk communication. They were trained during a mandatory session before the beginning of the evaluation period and meetings were organized regularly to clarify questions. To improve data quality and reduce observer bias, all teaching sessions were observed by two raters and one of the checklists was selected randomly. The raters pre-selected some classes for omission or did not complete checklists for some when they did not observe any risk communication topics being taught.

### Data processing

Checklists from both disciplines were separated according to teaching formats and different foci and objectives of the teaching sessions. Missing or incorrect information about courses and teaching formats was added after cross-checking with university class schedules. Checklists from courses designed as interdisciplinary courses rather than pure surgical topics were excluded. The checklists were analyzed to determine whether or not students were taught about communicating risks, what topics were used to teach them and how extensively were these topics explained. A secondary objective was to observe if the sessions were interactive and how often supplementary aids were used by lecturers.

Data were imputed using R (Version 3.3.2). Open text fields (comments) were analyzed descriptively. The datasets obtained and the research results did not include information on students and were based solely on the individual observations of the raters. The data were analyzed anonymously and no personal information of the raters was needed.

### Ethical approval

The Heidelberg University Medical School’s dean of studies was informed about the project and allowed the authors to approach and recruit students as participant observers for the study. Ethical approval was waived according to Paragraph 1 [1] of the constitution of University of Heidelberg medical faculty’s ethics committee under which supervision and ethical approval are only mandatory for research methods specifically with personal data directly related to human participants, the deceased and epidemiology projects. The raters were informed about the aims and objectives of the research in detail and consented to their participation in the form of contracts signed with the university hospital administration.

## Results

In total, the observers returned 326 checklists for 231 distinct teaching sessions (77% of all classes) with 95 duplicate checklists. The teaching sessions comprised lectures (n = 109), seminars (n = 77), practical courses (n = 3), Skills Lab (n = 11), PBL (n = 12), Medi-KIT (n = 8), Bed-Side Teaching (n = 9) and other teaching formats (n = 2). Checklists from 12 courses designated as interdisciplinary were excluded. The inter-rater reliability for duplicate checklists was calculated to be 81%. Of the 231 teaching sessions surveyed, 127 (84%, total 152) were from internal medicine and 104 (70%, total 149) were from surgery courses.

### Rate of prevalence

**Fig 1:**
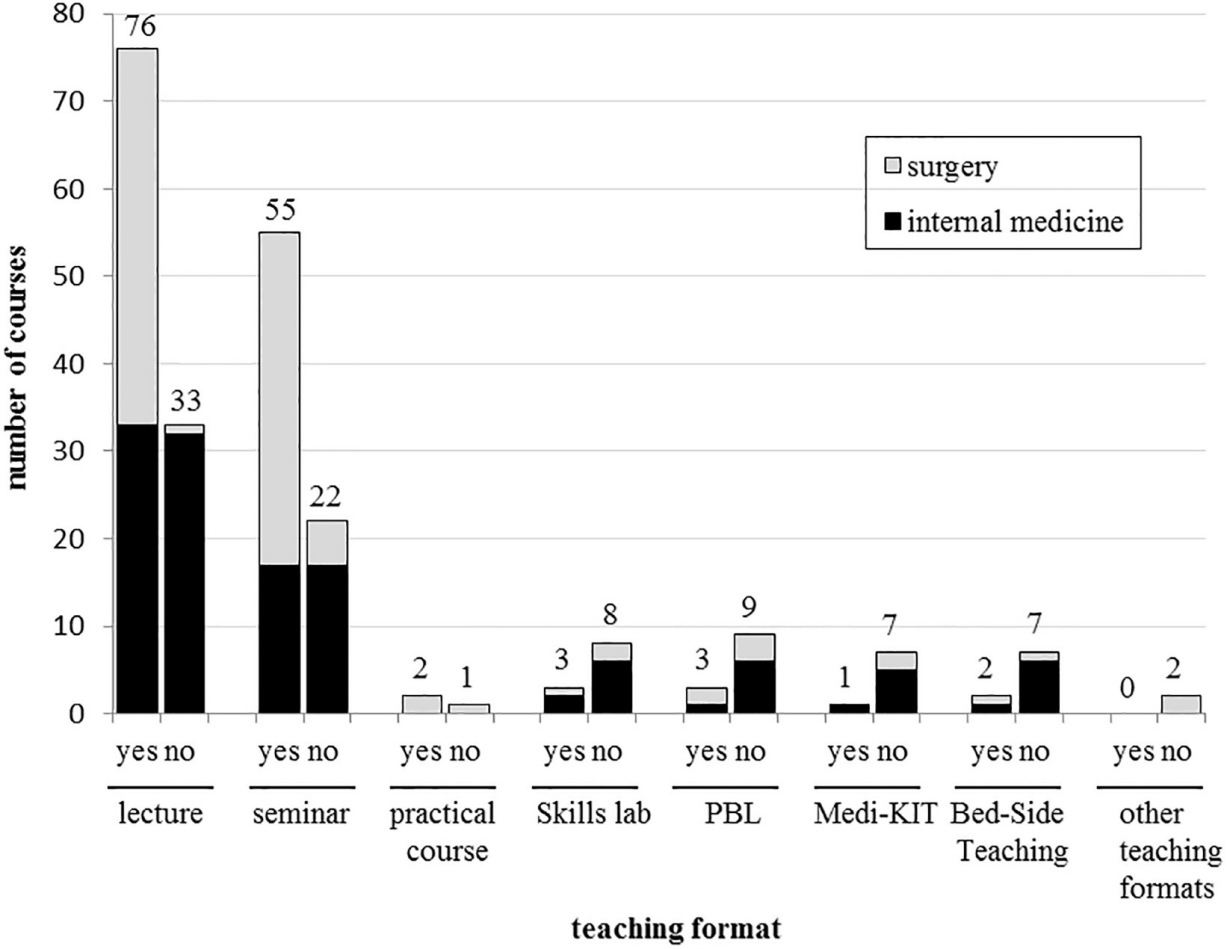
Prevalence of risk communication concepts in different teaching formats.

Students were taught about communicating risks in 61.5% (142/231) sessions. In internal medicine, the prevalence was 43.3% (n = 55) and 83.7% (n = 87) in surgery. Risk communication topics were 2.5 times more prevalent in large-group teaching formats (lectures 53.5% and seminars 38.7%) than in small-group settings. In both disciplines, the small-group formats rarely taught students about communicating risks (between 0% and 3.6%). Fig 1 summarizes the prevalence of risk communication teaching in each format at both departments.

### Prevalence of topics

The lecturers taught about concepts of risk communication using 197 topics during the classes. All topics (n = 197) were methodologically classified into 23 broader categories for ease of analysis with the objective of capturing teaching content rather than its numerical significance. The topics were condensed into 11 meta-categories on the basis of contextual or clinical similarities (Table 1). The topics that could not fit into any sub-category were coded as single meta-categories. Overlapping topics were multiply coded, for example lung cancer would be counted within ‘Cancer’ and ‘Lungs’ sub-categories.

**Table 1:** Topics divided into meta- and sub-categories according to conceptual and clinical contexts.

	Meta-category	Sub-category
Conceptual context	Therapy	Medication Therapy (non-pharmaceutical) Surgical operation
	Diagnosis	Clinical diagnostics X-ray images
	Prevention and prophylaxis	
	Patient safety	
	Emergency cases	
Clinical context	Specific diseases	Diabetes Rheumatism
	Organ systems	Joints and extremities Bones Pancreas and gall bladder Heart, vessels, ischemia and coagulation Liver Thyroid gland Esophagus, intestine and stomach Kidney, adrenal gland, bladder, urinary diversion and prostate Hematology Lungs Nerves and brain Psyche
	Infraction	Injuries Fractures
	Complications, wound and healing disorders	
	Pregnancy	
	Cancer	

**Fig 2:**
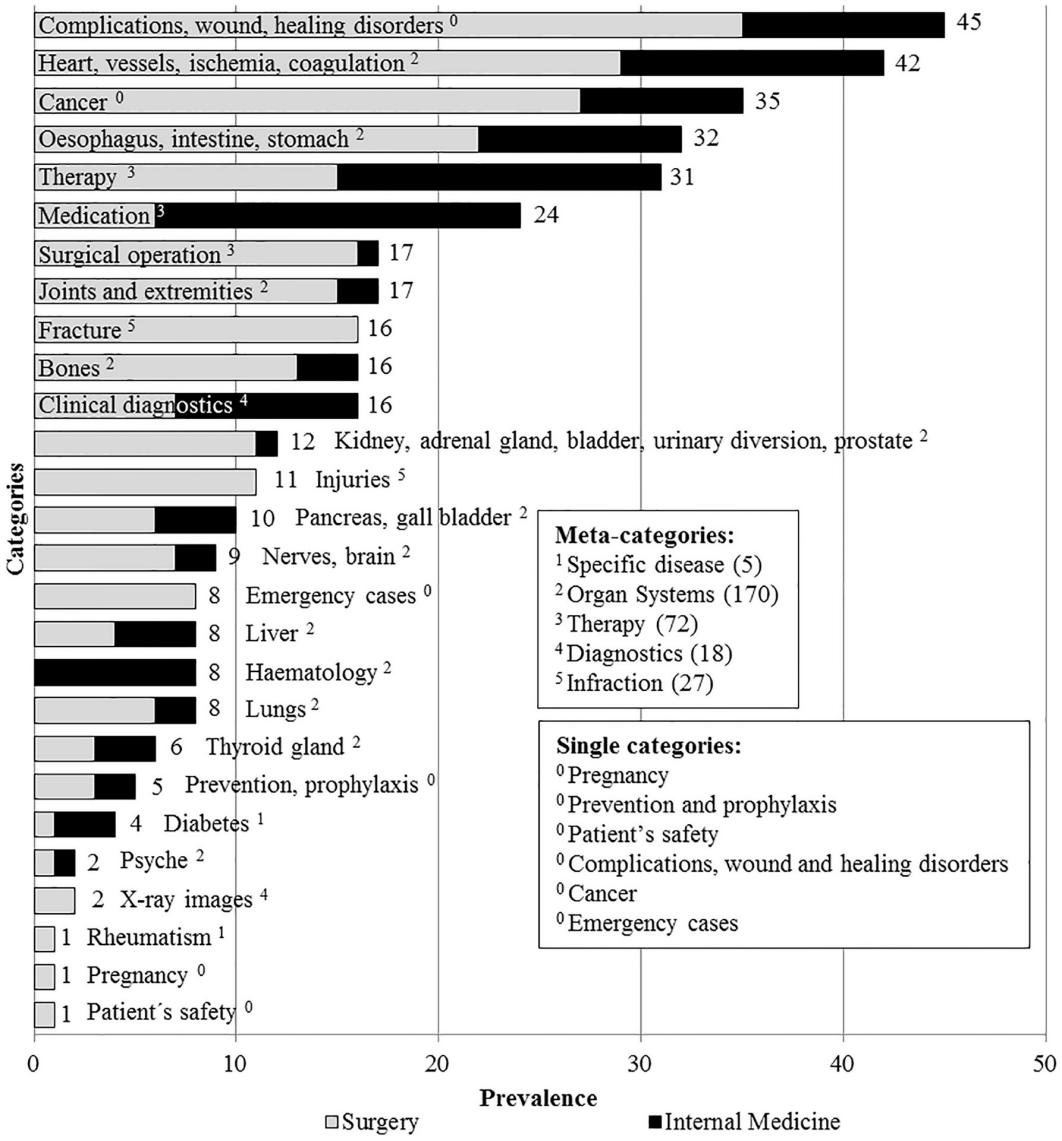
Prevalence of topics (merged into meta-categories) used to teach students on communicating risks.

The prevalence of different topics is summarized in Fig 2. For example, the meta-category ‘specific diseases’ comprising ‘diabetes’ and ‘rheumatism’ was used five times while ‘organ systems’ was used 170 times. Likewise, ‘cancer’ was used 35 times, whereas ‘prevention and prophylaxis’ were used only five times. Cancer of “esophagus, intestine, stomach” was discussed as a reference as often as all other cancers taken together.

### Teaching quality

Three key concepts of risk communication teaching namely “diagnostic measures”, “expected success” and “consequences” were taught most intensively during the sessions. The category “diagnostic measures” was discussed in 141 out of 142 sessions. The discussion intensity was rated ‘c’ or above (intensively taught) in 75.1% (n = 106) of the checklists. “Expected success” was discussed in 140 sessions and rated ‘c’ or above in 60.4% (n = 85). Similarly, “consequences” was rated ‘c’ or above in 60.4% (n = 85/141) of the sessions.

**Fig 3:**
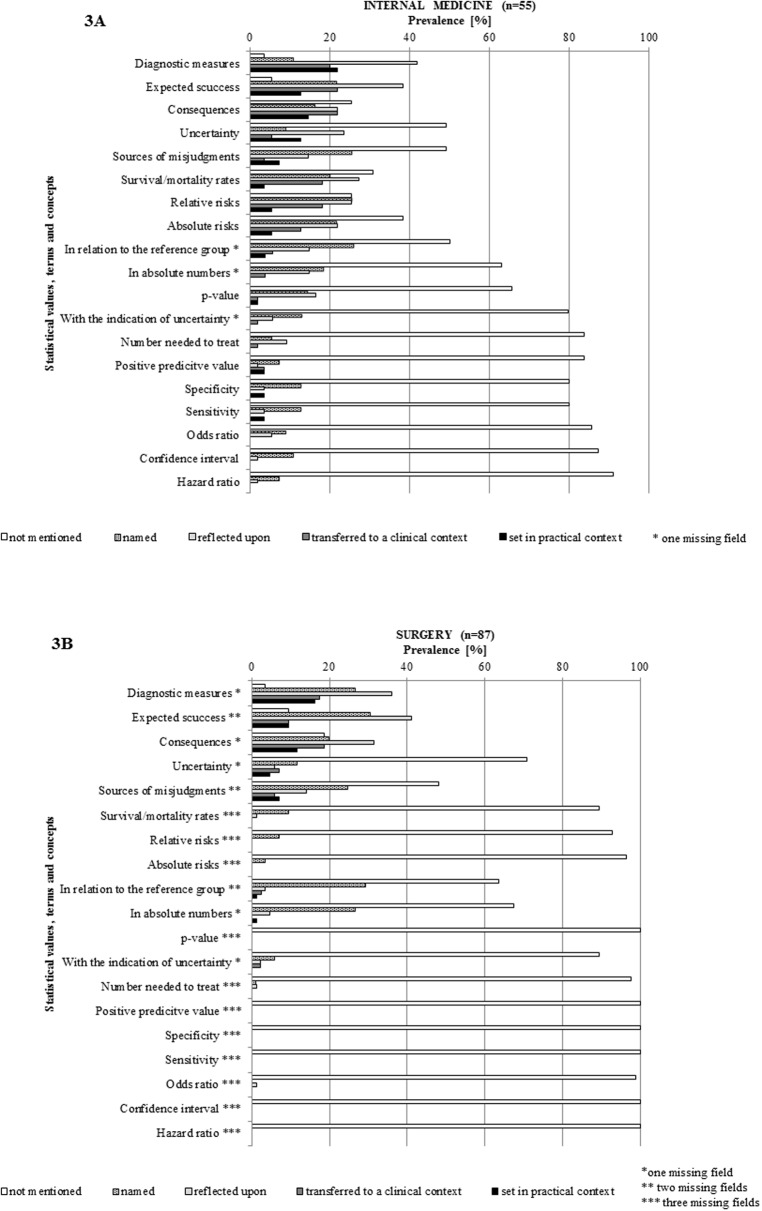
A and B: Frequency of statistical values, terms and concepts used by lecturers to teach about communicating risks.

These results are summarized in Fig 3A and 3B.

### Student engagement

On average, the lecturers allocated about 12 minutes (surgery = 7; internal medicine = 19) to teaching students on communicating risks. Questions and supplementary materials were noted as points of engagement during discussions at the teaching sessions. Students asked questions in 35 sessions (26.4%, surgery = 32; internal medicine = 3). Most questions were asked in large-group formats: lectures [18] and seminars [14].

Supplementary material was provided in 17 sessions (11.9%). Real patients (4 times), informative videos (3 times), handouts (once) and visual aid material such as X-ray photographs, a stoma pouch, pig skin to practice suture and a dummy to practice puncture (once each) were also used. Students rated them helpful in 95% of the cases.

## Discussion

This exploratory semester-long study of a medical curriculum provides an in-depth overview of the quantity and quality of risk communication teaching by triangulating data on prevalence, content, intensity and student-teacher interaction. Risk communication teaching is considered a prerequisite for effective physician-patient communication and failure of doctors to communicate inherent risks of medical treatments has been often blamed on insufficient teaching of risk quantification and biostatistics at medical schools. Although medical schools have in recent years integrated risk communication courses in curricula, literature on how and what is actually taught in the classrooms is limited. In line with previous studies, our results confirm that teaching quality of risk communication remains inadequate at medical schools. In the majority of teaching sessions surveyed for this study, lecturers did not teach risk communication using basic statistical values considered necessary for the interpretation of clinical data. This suggests an overall superficial teaching of risk communication by lecturers.

Higher prevalence of such topics in large-group seminars and lectures– 2.5 times more than small-group interactive sessions–highlights another problem of the theoretical approach of courses on statistics necessary for teaching risk communication. This is significant because generally lectures were not mandatory to attend and less than one-third of the enrolled medical students (48/164) attended these sessions in our study. For promoting a deeper understanding of risk communication among medical students, explanation of these statistical values in a clinical context is important. The students reported a higher level of learning when the lecturer not only talked about the topic but transferred it into a clinical context or a specific context of action. The other finding about students asking more questions in large-group teaching sessions, especially in surgery courses, was atypical since high-resource demanding, small-group teaching formats are recommended for interactive teaching topics such as risk communication. Teaching risk communication with the aid of clinical contexts and supplementary material can not only result in an interactive educational discourse but also promote a deeper understanding of the topics among students.

As may be expected, concepts of risk were referred to most frequently when talking about life-threatening health complications. Risk aspects were rarely discussed during teaching about other important topics such as prevention or prophylaxis. Professors often chose to teach students about risk communication with the example of cancer but frequently used the same type (e.g. esophagus, intestine, stomach) for discussion. For instance, lung cancer was used only three times to talk about communicating risks although it is considered to be the most preventable cancer type [46, 47]. This carries important public health implications, concerning treatment options versus preventive measures. Using examples of different forms of cancer could be more helpful for students not only to develop a broader understanding of risk communication but also for realizing that different cancer types might require different communication approaches.

Moreover, surgery students were taught about communicating risks more often although on a relatively superficial scale when compared to those in internal medicine, where more topics were taught on a practical level. Theoretically, internal medicine is a more “communicative” discipline whereas surgery is an “operative” discipline. For internal medicine doctors, therapy and side-effects of medicines may not always be directly linked with each other. On the other hand, post-operative complications carry serious consequences for the patient as well as the surgeon given the very nature of an operative intervention and the need for preoperative information consent of patients. Thus, teaching about communicating risks is equally important for students of both disciplines.

Internal medicine lecturers spent nearly three times more minutes on teaching students on communicating about risks. This may be one reason why few questions were asked by internal medicine students, suggesting adequate explanation by teachers as setting these concepts in a clinical context helped students to understand better. Moreover, transferring these concepts to a clinical or practical context was minimal although it is particularly important for creating a better understanding between patients and physicians. Students were rarely provided with supplementary material during the teaching sessions. However, they rated it helpful whenever it was provided. Supplementary material such as simulated patients can not only be useful in teaching about communicating risks in a clinical context, but can also be used to increase the interest of students in a particular subject.

A lack of interest by medical students in such topics may be a problem amidst a general underestimation about the serious impact such topics can have on health outcomes [48]. It remains an urgent matter of debate which concepts of risk communication should form a part of the medical curriculum and which clinical contexts should be used for this purpose. Some might favor topics that physicians have to deal with on a daily basis while others might prefer primarily life-threatening conditions. For example, teaching students to communicate risks by using examples of cancer can have positive consequences, considering more than 70% cases of 10 different cancer types with one specific risk factor are preventable [44].

Biostatistics terms such as hazard ratio or odds ratio are not only important aspects of risk communication for physicians during routine interactions with patients but are also useful for the patient to make informed decisions about medical interventions. Learning biostatistical values relevant to clinical epidemiology at school can help medical students to better understand risk communication, healthcare management as well as inherent risks identified within publications by pharmaceutical companies. Although it is practically impossible to teach students about all statistical concepts about all diseases, the absence of teaching on even the basic biostatistics terms in the majority of classes suggests a much deeper problem. Previous studies have reported on the inability of physicians when it comes to understanding and using statistical information [5, 25, 47, 49–53]. Statistical illiteracy has been cited as a common problem in misinterpreting the harms and benefits of diagnostic or therapeutic interventions or inability to explain absolute or relative risk reduction [11, 49, 51]. Thus, understanding statistical data is of utmost importance for doctors seeking to adequately explain healthcare data and procedures to patients. Studies have also found a lack of statistics courses in medical curricula, inadequate statistical knowledge among professional doctors and limited didactical expertise of lecturers at universities [48, 54].

Teaching medical students about communicating risks is gradually gaining relevance and becoming a more regular part of medical education all over the world [55, 56]. New teaching approaches and innovative formats are being developed to reach a higher knowledge level that prepares students for their future responsibilities [57]. Our findings will be useful not only for medical educators to gain a better understanding of the characteristics of risk communication teaching but also for medical schools and curriculum planners to identify existing gaps in medical education.

## Conclusion

The importance of risk communication for medical professionals as an underestimated factor for patients’ health outcomes cannot be emphasized enough at a time when shared decision-making and informed consent are gaining increasing importance. This observational study audited a medical curriculum for the prevalence and quality of teaching on risk communication at a medical school. Our results confirm a profound lack of core statistical concepts necessary for the interpretation of clinical data in the teaching sessions. Even when these values were taught, their discussion stayed at a superficial level whereby lecturers rarely went to the stage of transferring the concepts to a clinical example. This was confirmed by the much higher prevalence of such topics in large-group theoretical seminars and lectures than small-group practical, interactive sessions. Moreover, topics of teaching risk communication were found to be saturated within several categories of diseases such as cancer and complications, and inclusion of more diversified topics as well as preventive measures could prove to be beneficial for medical students. Finally, providing medical students with supplementary teaching material such as clinical reports or simulated patients has proven extremely useful and a thus a variety of different materials such as handouts or digital blended-learning formats could also be utilized.

Although this might be influenced by the teaching styles of individual lecturers and not the entire curriculum, our results suggest that medical schools need a larger and more profound representation of courses to teach students on communicating risks to patients with special focus on knowledge transfer through practical, interactive exercises in a clinical context. Given the limited literature on this topic, an extensive study encompassing more medical disciplines, possibly using more participant observers, would be highly recommended for future research, not only for identifying knowledge gaps and practical implications but also for developing future policies on medical curricula.

## Supporting information

S1 Dataset (XLSX)
